# The effects of dynamic daylight-like light on the rhythm, cognition, and mood of irregular shift workers in closed environment

**DOI:** 10.1038/s41598-021-92438-y

**Published:** 2021-06-22

**Authors:** Jingxin Nie, Tianhang Zhou, Zhizhong Chen, Weimin Dang, Fei Jiao, Jinglin Zhan, Yifan Chen, Yiyong Chen, Zuojian Pan, Xiangning Kang, Yongzhi Wang, Qi Wang, Yan Tang, Wentian Dong, Shuzhe Zhou, Yantao Ma, Xin Yu, Guoyi Zhang, Bo Shen

**Affiliations:** 1 State Key Laboratory for Artificial Microstructure and Mesoscopic Physics, School of Physics, Peking University, 209, Chengfu Road, Haidian District, Beijing, 100871 China; 2 Peking University Sixth Hospital, Peking University Institute of Mental Health, Key Laboratory of Mental Health, Ministry of Health (Peking University), Haidian District, 51, Huayuan North Road, Beijing, 100191 China; 3 State Key Laboratory of Nuclear Physics and Technology, School of Physics, Peking University, Beijing, 100871 China; 4 Dongguan Institute of Optoelectronics, Peking University, Dongguan, 523808 Guangdong China; 5 Department of Physical Education, Peking University, Beijing, 100871 China

**Keywords:** Physiology, Health care, Optics and photonics

## Abstract

Shift workers are mostly suffered from the disruption of circadian rhythm and health problems. In this study, we designed proper light environment to maintain stable circadian rhythm, cognitive performance, and mood status of shift workers. We used five-channel light-emitting diodes to build up the dynamic daylight-like light environment. The illuminance, correlated color temperature, and circadian action factor of light were tunable in the ranges of 226 to 678 lx, 2680 to 7314 K, and 0.32 to 0.96 throughout the day (5:30 to 19:40). During the nighttime, these parameters maintained about 200 lx, 2700 K, and 0.32, respectively. In this light environment, three subjects had engaged in shift work for 38 consecutive days. We measured plasma melatonin, activity counts, continuous performance tests, and visual analogue scale on mood to assess the rhythm, cognitive performance, and mood of subjects. After 38-day shift work, the subjects’ peak melatonin concentration increased significantly. Their physiological and behavioral rhythms maintained stable. Their cognitive performance improved significantly after night work, compared with that before night work. Their mood status had no significant change during the 38-day shift work. These results indicated that the light environment was beneficial to maintain circadian rhythm, cognitive performance and mood status during long-term shift work in closed environment.

## Introduction

Nowadays, about 20% of workers in the world are arranged to shift work, including early morning work, night work, rotating shift work, and irregular shift work^1,2^. The shift workers are required to disrupt the regular sleep/wake cycle, which has negative effects on the sleep quality, cognition, and mood status^3–10^. For several days or weeks of shift work, the circadian rhythm would be disrupted^11–13^ and the risks of many chronic diseases would increase, such as diabetes, cancer, metabolic diseases, and chronic mood disorder^3,11,14^. Therefore, it is necessary to take some countermeasures to improve work performance and decrease health problems of shift workers. Many previous studies recognize light environment as one of the most significant factors to solve health problems of shift workers^4,5,11–19^. The bright light of about 900 to 5000 lx is used to reset the circadian phase and promote alertness^20–22^. Besides the illumination, the duration and the timing of light exposure are also associated with sleep quality, cognitive performance, and circadian rhythm^12,16,23^.

The intrinsically photosensitive retinal ganglion cells (ipRGCs) are sensitive to the blue light and play an important role in regulating the circadian rhythm^24,25^, so the spectrum of light is also significant in the light environment. Blue-enriched or high correlated color temperature (CCT) light improves alertness and work performance^5,13,16,26,27^. However, blue light also suppresses the melatonin secretion during night work, which easily results in the circadian disruption, depression, and blue light hazard^18,28^. Some researchers use the short-wavelength attenuated white light at night to maintain the melatonin concentration, combined with no deleterious effects on sustained attention and subjective alertness^4,11,18^. However, the color discrimination capability significantly decreases under the short-wavelength attenuated white light at night, so high color fidelity is also required for the light at night. High color fidelity is also beneficial to the visual comfort and color perception^29^. Therefore, it is significant to investigate and optimize the spectrum of light environment to benefit the shift workers.

Most shift workers undergo the misalignment between the internal circadian rhythms and the imposed work/rest schedules^8,12,13,17,26^. The circadian phase can be reset by bright light or blue light at certain time^12,13,26^. However, in some cases, the periods of work/rest schedules are deviated a lot from 24 h, such as 18 h^16^ and 28 h^30^. In these cases, the circadian rhythm can hardly be entrained by the external work/rest schedules. And resetting the circadian phase of shift workers is impossible for long-term shift workers^17,30,31^. The free-running period of human circadian rhythm is in the range of 24.2 to 24.7 h and has tens of minutes deviation from 24 h^30–32^. The deviation has negative effect on the shift workers’ performances in some closed environments, such as space stations or submarines^16,17^. Therefore, an effective zeitgeber is required to entrain the circadian rhythm of workers in closed environments. The spectrum and illuminance of the natural daylight change periodically with 24-h period, so the natural daylight is regarded as a zeitgeber to maintain the circadian rhythm^29,33^. However, most artificial light sources maintain the constant spectrum and illuminance. The dynamic daylight-like light-emitting diodes (LEDs), which simulate the natural daylight with time-dependent spectrum and illuminance, have some positive circadian effects^29,34–38^. Some lighting designers propose circadian action factor (CAF) to assess the circadian effects of light sources^34,39,40^. They believe the tunability of CAF to be the larger the better, because the higher CAF during the daytime enhances the vigilance and excitation, and then potentially improves work efficiency of workers. The lower CAF means less melatonin suppression and benefits the sleep quality at night, referring to previous studies^34,41^. However, some designers do not conduct biological experiments to investigate the circadian effects of these light sources. Considering the individual differences in the responses to the light, the actual effects of the light sources on the humans should be investigated in the biological experiments^42^. Some biological experiments are conducted only during the daytime or nighttime, while the light exposure before the experimental time might influence the circadian rhythm and alertness in the experiment^28,43,44^, so a closed environment is necessary to control the light environment all the time^33^. In addition, the non-visual effects of light gradually occur with the increasing number of consecutive days^15^, and approximately one month is sufficient to establish stable circadian rhythm^30^, so the shift work in closed environment is designed to be more than one month in this study.

The biological effects of the light environment on the shift workers were reported in many previous studies^4,5,7,11,12,16–19,29,36^. In this study, we built up dynamic daylight-like light environment and aimed to maintain stable circadian rhythm, benefit cognitive performance and mood status of shift workers for approximately one month in the closed environment. The five-channel LEDs, consisted of red, green, blue, warm white, and cool white (RGBWW) five chips, were fabricated to build up the dynamic daylight-like light environment. The CAF, CCT, and illuminance of the dynamic daylight-like light were designed to simulate the natural daylight during the day. These parameters were tunable in the ranges of 226 to 678 lx, 2680 to 7314 K, and 0.32 to 0.96 throughout the day (5:30 to 19:40). In the forenoon (5:30 to 12:00), these parameters increased and they decreased in the afternoon (12:00 to 19:40). The CAF, CCT, and illuminance fixed as approximately 0.32, 2700 K, and 200 lx at night. Three subjects engaged in irregular shift work in the closed environment for 38 consecutive days. They were arranged in three different types of shift work schedules every three days. During the experiment, the performances of the shift workers were evaluated from many aspects. The plasma melatonin was measured to assess the physiological rhythm^5,11,12,16–18,41^. The wrist-activity monitors recorded the daily activity counts to reflect the behavioral rhythm^8,11,28,45^. The cognitive performance was tested by continuous performance test (CPT)^5,6^. The mood status was assessed by visual analogue scale (VAS)^29,46^. At last, the relationships between the performance of shift workers and the parameters of light sources were discussed.

## Results

### Plasma melatonin

**Figure 1:**
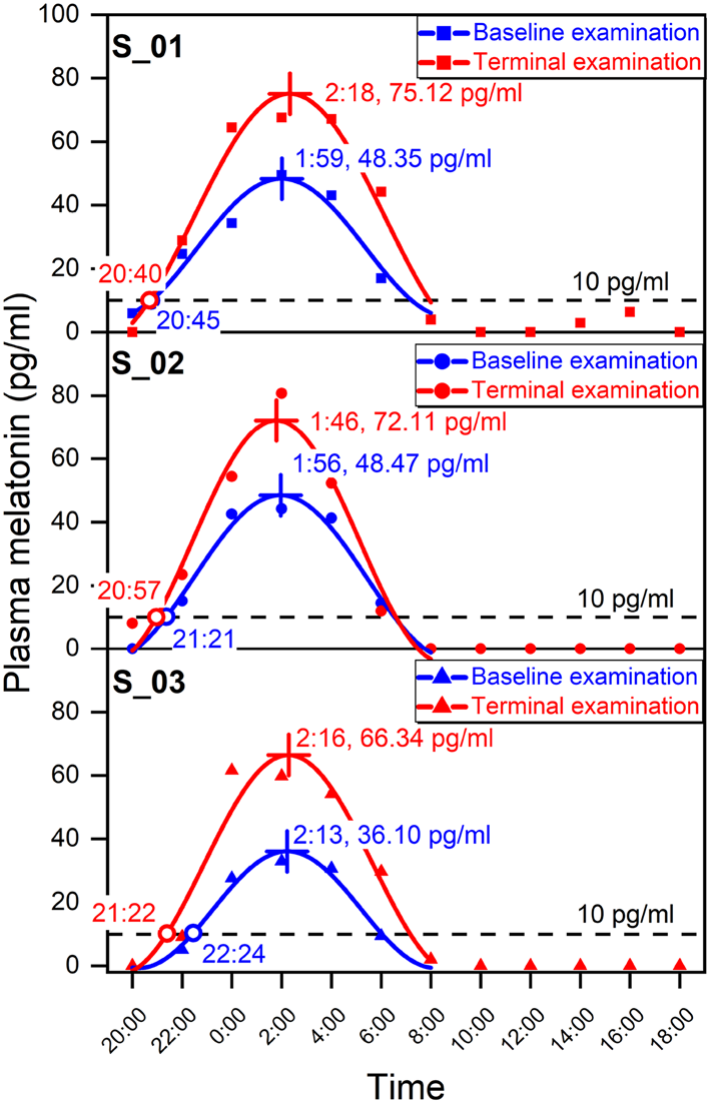
The plasma melatonin concentrations and their cosine fitting functions for three subjects. The symbols represent the measured melatonin concentrations. The lines are the cosine fitting functions of these points. The blue points and lines represent the melatonin patterns in the baseline examination (D7 20:00–D8 6:00). The red points and lines represent the melatonin patterns in the terminal examination (D46 20:00–D47 18:00). (The figure was generated by Origin 2018, URL: https://www.originlab.com/).

We measured the plasma melatonin two rounds to evaluate the subjects’ circadian rhythm during the experiment. The baseline examination (D7 20:00-D8 6:00) was conducted at the beginning of living in the closed environment, while the terminal examination (D46 20:00-D47 18:00) was at the end of shift work. Figure 1 shows the subjects’ plasma melatonin patterns in the two examinations. The coefficients of determination (CODs) of the fitting functions are all above 0.95, indicating cosine function is a good fitting model. S_01, S_02, and S_03 represent three subjects in the experiment. After 38 consecutive days of shift work, the phase shifts of the peak melatonin times were 19.23, -9.99, and 3.03 min for S_01, S_02, and S_03, respectively. There was no statistically significant between the baseline and terminal examinations (df = 2, t = 0.48, *p* = 0.676). The subjects’ peak melatonin concentrations increased significantly from 48.35, 48.47, and 36.10 pg/ml to 75.12, 72.11, and 66.34 pg/ml (df = 2, t = 14.10, *p* = 0.005) after 38-day shift work. Notably, in the baseline examination, the melatonin onset times for the subjects were in the range of 20:45 to 22:24, and the time difference was nearly 100 min. In contrast, in the terminal examination, the melatonin onset times ranged from 20:40 to 21:22, and the time difference was 42 min. The standard deviation (SD) of subjects’ melatonin onset times also decreased from 40.91 min to 17.25 min. These results indicated the melatonin onset time tended to be similar in the terminal examination.

### Activity counts

The wrist-activity monitors recorded the activity counts every minute, which reflected the behavioral rhythm of subjects. The interdaily stability (IS), intradaily variability (IV), and relative amplitude (RA) at the beginning and the terminal of the experiment were calculated according to the activity counts, as shown in Table 1. Comparing the first three days (D15-D17) with the last three days (D47-D49), the IS, IV, and RA presented no significant differences (*p* > 0.05). These three nonparametric variables also showed no significant differences (*p* > 0.05) between the first 6 days (D15-D20) and the last 6 days (D44-D49). The Z and p values of Wilcoxon signed-rank tests were shown in Table 1.

**Table 1:** The comparison of IS, IV, and RA at the beginning (D15-D20) and terminal (D44-D49) of the experiment.

Day	IS			IV			RA		
	S_01	S_02	S_03	S_01	S_02	S_03	S_01	S_02	S_03
D15	0.280	0.401	0.258	0.907	0.653	0.698	0.827	0.752	0.917
D16	0.147	0.159	0.451	0.722	0.537	0.427	0.848	0.807	0.737
D17	0.193	0.404	0.278	0.969	0.448	0.701	0.721	0.874	0.894
D18	0.375	0.404	0.490	0.565	0.443	0.417	0.902	0.847	0.947
D19	0.414	0.447	0.341	0.458	0.428	0.496	0.906	0.883	0.821
D20	0.241	0.397	0.325	0.966	0.739	0.659	0.644	0.801	0.895
D44	0.344	0.297	0.311	0.708	0.594	0.717	0.664	0.836	0.970
D45	0.380	0.431	0.297	0.601	0.587	0.577	0.950	0.778	0.946
D46	0.364	0.323	0.373	0.633	0.387	0.575	0.853	0.884	0.891
D47	0.259	0.223	0.229	0.897	0.611	0.563	0.700	0.726	0.884
D48	0.393	0.357	0.280	0.546	0.429	0.646	0.946	0.747	0.931
D49	0.334	0.310	0.300	0.627	0.581	0.653	0.920	0.618	0.909

Wilcoxon signed-rank tests	Z	p		Z	p		Z	p	
First and last 3 days	− 0.118	0.910		1.066	0.301		0.118	0.910	
First and last 6 days	0.436	0.671		0.305	0.760		-0.174	0.862	

### Continuous performance test

**Figure 2:**
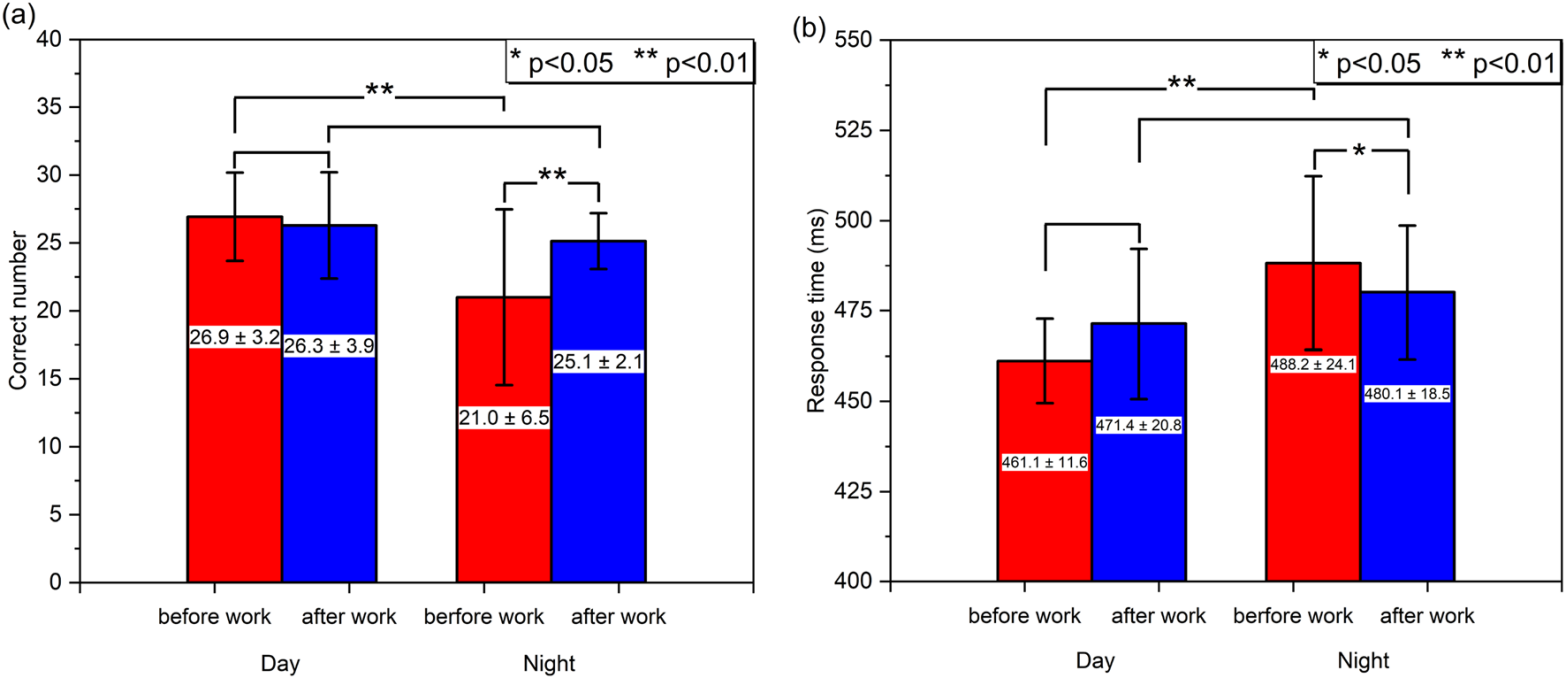
(**a**) The number of correct responses and (**b**) response time of CPT after and before working segment during the daytime and at night. The red and blue columns represent the CPT results before and after work, respectively. The mean ± SD is marked on the columns and the error bars represent the SD. (The figure was generated by Origin 2018, URL: https://www.originlab.com/).

After approximately one month of shift work, the subjects were required to perform CPTs before and after their working segments. The number of correct responses and response time in the CPT reflected subjects’ sustained attention and cognition, as shown in Fig. 2. As for day work, the number of correct responses decreased a little from 26.9 ± 3.2 (mean ± SD) to 26.3 ± 3.9 after work than before work (df = 13, t = 0.71 and *p* = 0.493), and the response time increased from 461.1 ± 11.6 ms (ms) to 471.4 ± 20.8 ms (df = 13, t = 2.11 and *p* = 0.055), but the difference was not significant. In contrast, after working at night, the number of correct responses increased significantly from 21.0 ± 6.5 to 25.1 ± 2.1 (df = 22, t = 2.98, *p* = 0.007), and the response time decreased significantly from 488.2 ± 24.1 ms to 480.1 ± 18.5 ms (df = 22, t = 2.59, *p* = 0.017). We also compared the cognitive performance in the daytime and at night. Before work, the number of correct responses during the nighttime was significantly worse than the daytime (df = 35, t = 3.18 and *p* = 0.003), and the response time also showed the similar results (df = 35, t = 3.93 and *p* = 0.0004). However, after work, the number of correct responses between the nighttime and the daytime showed no significant difference (df = 35, t = 1.18, *p* = 0.246). The response time also had no significant difference (df = 35, t = 1.33, *p* = 0.194).

### Visual analogue scale (VAS) on mood

**Figure 3:**
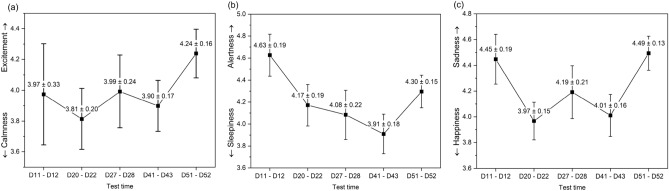
Subjects’ mood values in five assessments during the experiment (mean ± SEM), including (**a**) calmness/excitement, (**b**) sleepiness/alertness, and (**c**) happiness/sadness. The first assessment was before the shift work. The last assessment was after the shift work. The other three assessments were during the consecutive shift work. (The figure was generated by Origin 2018, URL: https://www.originlab.com/).

We evaluated the mood of subjects by three kinds of VAS on mood, including calmness/excitement, sleepiness/alertness, and happiness/sadness. We performed five sessions of assessments during the experiment. The mean and standard error of mean (SEM) of each assessment are shown in Fig. 3. According to repeated analysis of variance (rANOVA), there were no significant difference of all the three kinds of mood status between five times mood assessments (df = 4, F = 0.61 and *p* = 0.656 for calmness/excitement, F = 1.82 and *p* = 0.126 for sleepiness/alertness, F = 2.21 and *p* = 0.068 for happiness/sadness).

## Discussion

The circadian rhythm was evaluated by plasma melatonin and activity counts. The plasma melatonin was the physiological parameter about the circadian rhythm, and activity counts reflected the behavioral rhythm. The similar peak melatonin times in the two examinations demonstrated that the physiological rhythm maintained stable after long-term shift work in the closed environment^41^. Previous studies reported that the significantly increasing melatonin concentration at night was beneficial to the sleep quality^34,41^. The increasing melatonin at night also meant the increasing amplitude of circadian rhythm, indicating the strength of circadian rhythm was enhanced^47^. The decreasing difference of melatonin onset times of three subjects indicated the entrainment effect on the circadian rhythm. The subjects’ behavioral rhythm during the experiment could be assessed by IS, IV, and RA. IS, IV, and RA respectively reflected the strength of coupling of rhythm to light environment, the fragmentation of activity/rest, and the relative strength of activity variation during the day^45^. The three parameters showed no significant differences between the beginning and the terminal of shift work, indicating subjects’ behavioral rhythm maintained stable during long-term consecutive shift work.

**Figure 4:**
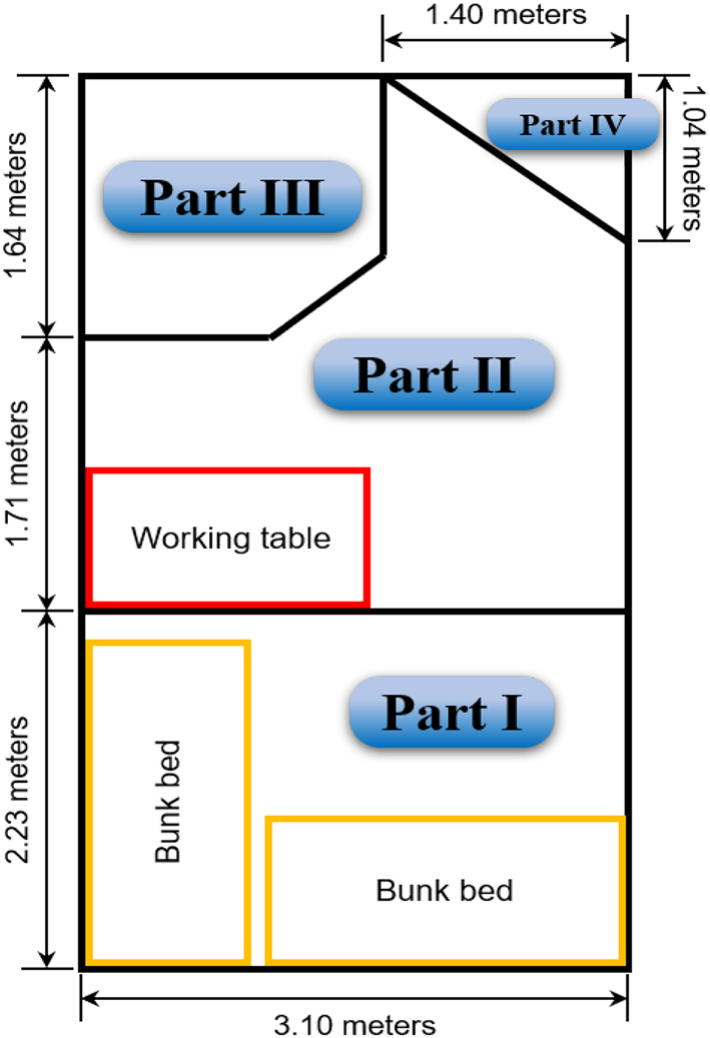
The schematic diagram of the four partitions in the experimental ward. The closed experimental environment contains Parts I, II, and III. (The figure was generated by PowerPoint 2019, URL: https://www.microsoftstore.com.cn/software/office).

The subjects’ cognitive performance improved significantly after night work than before night work, while there was no obvious change after working in the daytime. Also, cognitive performance before work at night was significantly poorer than other times, indicating the light environment and/or night work significantly improved cognitive performance. As shown in Fig. 4, Parts I and II were the rest area and work area, respectively. The subjects worked in Part II where the dynamic daylight-like light was always on. Out of working time, they tended to keep the light on during the daytime, while turn it off at night in Part I. The similar cognitive performance before and after work during the daytime indicated that the effects of the light environment and/or day work on cognition were not significant. As for night work, subjects were usually in dark environment before work, which could lead to poor cognitive performance before night work. The sleep inertia potentially led to the poor cognition before night work as well^48^. After night work, the cognitive performance improved significantly and recovered to the level in the daytime. It could be explained that white light with low CAF and CCT at night was beneficial to cognitive performance, compared with dark environment. The effects of sleep inertia on the cognition was not significant after waking up 20 to 30 min^48,49^, while the time interval between waking up and CPTs was more than 20 min in most cases in this study. Therefore, the white light with low CAF, CCT, and illuminance primarily contributed to the improvement after night work. There was little proportion of short wavelength in the approximately 2700 K white light at night. Previous studies found that the short wavelength attenuated white light could maintain sustained attention and cognitive performance without obvious suppression of melatonin^4,18^. Shift workers usually suffered from poor cognition at night^3^. In contrast, in this study, the subjects’ cognitive performance at night could recover to the level during the daytime. Meanwhile, their plasma melatonin indicated the circadian rhythm was stable and its strength was enhanced after the 38-day shift work. These results demonstrated that the light environment benefited the cognitive performance during the nighttime, without disturbance of melatonin rhythm.

The similar mood status indicated that the subjects’ mood status was stable in the five assessments. In Fig. 3a, the values on the calmness/excitement scale were stable between 3.82 and 4.24 in the first four assessments. In the last assessment when the subjects finished the long-term shift work, an increase was in the direction of excitement. As for the sleepiness/alertness scale, there was an increase in the direction of sleepiness when the consecutive shift work processed approximately one month, as shown in Fig. 3b. The little decreasing alertness could be due to the increasing melatonin concentration at night^50^. However, according to the above discussions, the cognitive performance and circadian rhythms maintained well, indicating the little decreasing alertness had no negative effect on the work performance. Figure 3c shows the happiness/sadness mood in the assessments. There was an increase in the direction of happiness on the happiness/sadness scale in the middle three assessments, indicating subjects’ happiness increased when they were in the consecutive shift work. The happiness mood could be related to the stable circadian rhythm and/or their food, physical exercises, and health. For shift work or living in the closed environment, it was easy to suffer from bad mood because of the disruption of circadian rhythm, the decreasing work performance, and improper light at night^7,14^. However, in this study, the dynamic daylight-like light during the daytime was helpful to entrain the circadian rhythm, and the white light with little proportions of short-wavelength at night benefited the cognitive performance without disturbance of rhythm. The light environment with high level of CRI was beneficial to visual comfort and mood^29^. All these factors of the light environment resulted in the stable mood status during the experiment.

According to the present study and previous studies, we concluded the relationships between the parameters of light sources and the performances of shift workers. The dynamic illuminance, CCT, and CAF during the daytime which simulated the natural daylight were helpful to entrain the circadian rhythm^30,51^, according to the stable peak melatonin times and no significant change of IS, IV, and RA during the experiment. The amplified tunability of CCT and CAF resulted in the increasing melatonin at night, which was beneficial to sleep quality and was regarded as enhancing the strength of rhythm^34^. The white light with proper illuminance and at low CCT and CAF at night was supposed to maintain sustained attention and cognition, without disturbance of melatonin^4,18^. As we expected, there was little phase shift after 38-day shift work with more melatonin concentration at night, and the cognitive performance after night work recovered to the level in the daytime. Also, the optimal CRI and white light at low CCT at night were beneficial to mood status when living in the environment with long-term artificial lighting^29^. However, the limited experimental cost resulted in the small number of subjects and the lack of control groups, which resulted in the limited practicality because of individual differences in the responses to the light^42^. This study limited the gender, age, and work experience of shift workers. The type of shift work, social relationship, and work space might also have effects on the shift workers in the closed environments. Therefore, the effects of the dynamic daylight-like light on the rhythm, cognition, and mood could be further investigated on more subjects and environments. The experimental cost would be reduced by cooperating with more and more users who adopt the dynamic daylight-like light sources.

In summary, a dynamic daylight-like light environment was constructed by RGBWW LEDs. The three subjects had engaged in shift work in this closed light environment for 38 days. The dynamic daylight-like light provided time-dependent illuminance, CCT, and CAF with high CRI values, referring to the natural daylight. After consecutive 38-day shift work, subjects’ plasma melatonin and activity counts indicated that the circadian rhythm maintained stable and its strength was enhanced. The CPT and plasma melatonin demonstrated that the light at approximately 2700 K benefited the cognition at night, without disturbance of melatonin rhythm. The VAS mood assessments illustrated the mood status was stable during the long-term shift work. Consequently, the results above indicated that the dynamic daylight-like light environment for shift workers effectively entrained and enhanced the strength of circadian rhythm, combined with good cognition and mood.

## Methods

### Subjects and work schedule

We selected the subjects in the nearly same age and the same gender to decrease the effects of individual differences. On average, the age of shift workers was usually in the range of 30 to 45 years^15,52,53^, so we selected the subjects in that range with more than 5 years of shift work experience. Also, considering the physical activity, males were easier to be influenced in the long-term isolation^54^, so we selected male subjects. Three male subjects (aged 39, 40, and 42) volunteered to participate in the experiment. All participants gave written informed consent. They were randomly numbered as S_01, S_02, and S_03. The human experiment was approved by the Ethics Committee of Peking University Institute of Mental Health (No. 2018-12-26-5), and it was also performed in accordance with the Declaration of Helsinki.

The closed environment was transformed from a hospital ward in Beijing by covering all the windows and doors with light-tight boards and black papers. The ward was divided into four partitions, as shown in Fig. 4. Part I was the rest area for sleep and rest. There were two bunk beds in it. Part II was the work area where subjects performed shift work, had meals, and did exercises at regular times. Part III was the toilet and bathroom. Part IV was the exchange area for exchanging food, fruit, water, and rubbish.

**Figure 5:**
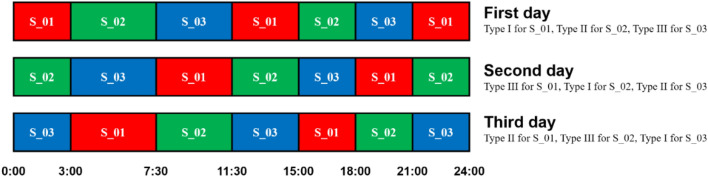
Shift work schedule of three subjects in a three-day cycle. The red, green, and blue areas represent the working segments of subjects S_01, S_02, and S_03, respectively. Each subject work 7 segments every three days, consisted of one Type I day, one Type II day, and one Type III day. (The figure was generated by PowerPoint 2019, URL: https://www.microsoftstore.com.cn/software/office).

In the first 7 days (D1–D7), subjects lived in the ward at night, and they could go around at the hospital freely during the daytime to adapt to the environment. They were introduced the working tasks and trained for the wrist-activity monitors in the experiment. They were also told to prepare for the new time zone in the next four days. They were encouraged to sleep and get up late. After that, from D8 to D49, subjects were required to live in the closed environment (Parts I, II, and III) all the time. The time in the closed environment delayed for 4 h. That was to say, the local time of Beijing was in GMT + 8:00 time zone, while the closed environment was in GMT + 4:00 time zone. This action was designed to avoid the disturbance of subjects’ intrinsic circadian rhythm, observers’ behaviors, and some other factors, such as the temperature and noise in the environment. Subjects had to establish new circadian rhythm under the experimental conditions. Subjects could adapt to the new time zone and the closed environment in the next 4 days (D8–D11). In these four days, subjects worked from 8:00 to 18:00 during the daytime and slept at night. From D12, subjects began to take shift work in turn until D49. The shift work schedule referred to their shift work experiences. During the 38 consecutive days (D12-D49), there were 7 working segments each day, including 0:00–3:00, 3:00–7:30, 7:30–11:30, 11:30–15:00, 15:00–18:00, 18:00–21:00, and 21:00–24:00, as shown in Fig. 5. One subject was required to work and the other two subjects could rest or sleep at any time. Subjects were asked to solve sudoku, mazes, math problems and fulfill recitation, transcribing, and reading tasks in the working time. The shift work schedule is shown in Fig. 5. The subjects worked two or three segments in one day and worked 7 segments every three days. There were three types of days according to the working times in one day. Working at 0:00–3:00, 11:30–15:00, and 21:00–24:00 was called as Type I. Working at 3:00–7:30 and 15:00–18:00 was called as Type II. Working at 7:30–11:30 and 18:00–21:00 was called as Type III. Each subject experienced one Type I day, one Type II day, and one Type III day in a three-day period. Therefore, three days were regarded as a cycle in the shift work. After 38-day shift work, the ward was open, and subjects lived in it for another 7 days (D50–D56). After the physical and psychiatric examinations confirmed that all the subjects were healthy, the experiment ended.

### Light environment

In the light-tight ward, Parts I and II were equipped with the same RGBWW LEDs on the ceiling (Shengdi, Dongguan, China), replacing the fluorescent lamps. The light primarily illuminated the working table and subjects vertically. The observers’ eyes were approximately 35 cm above the working table, and the illuminance from the observers’ point of view was about 1.5 times than it on the working table. Part III was equipped with a red light at approximately 630 nm, which was minimally sensitive to ipRGCs and had little non-visual effects^25^. The light sources in Part II were always on during the experiment because there was one subject working all the time. The subjects could turn off the light sources in Part I when they slept. They could turn on the light in Part III when they went to toilet or took a bath.

**Figure 6:**
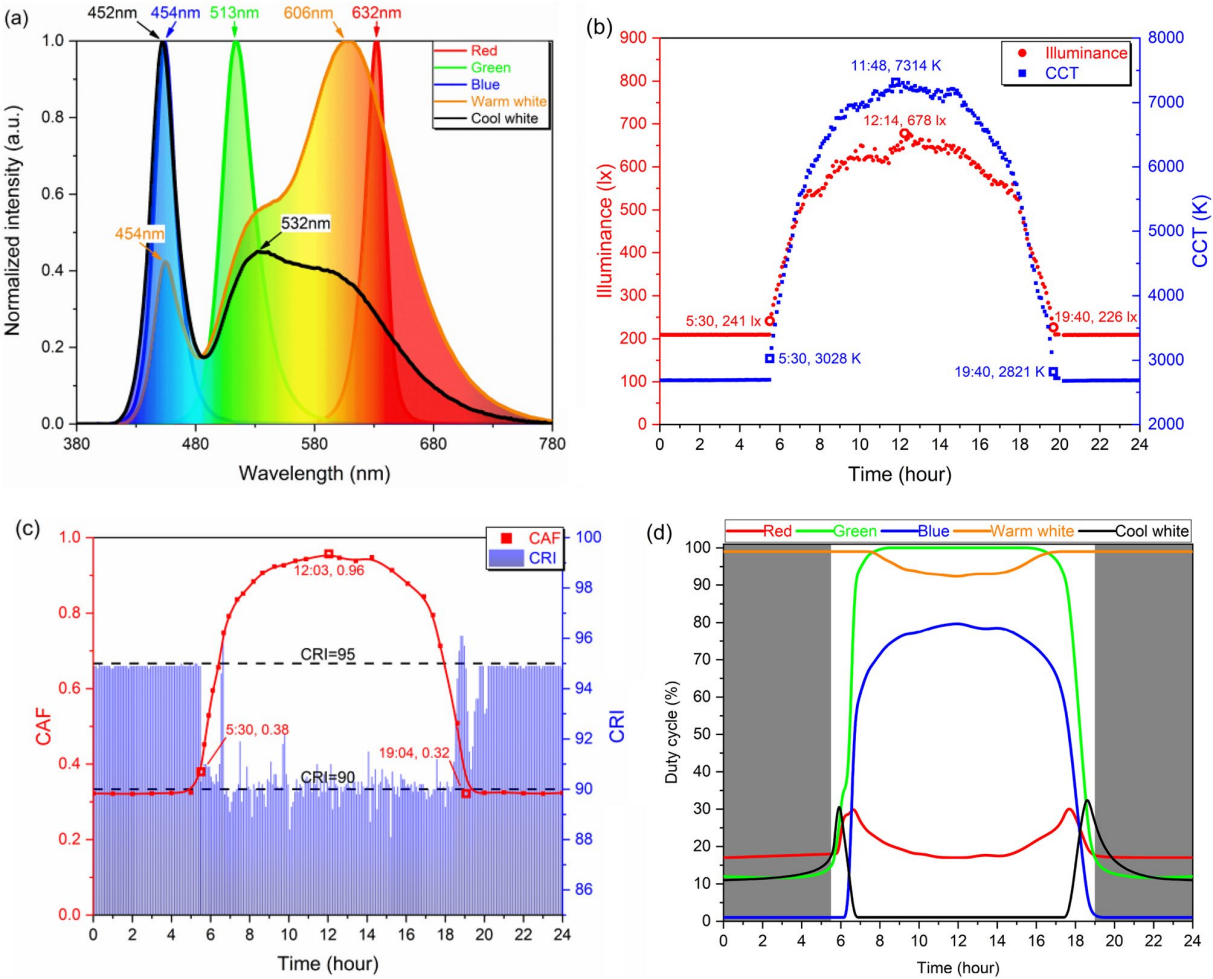
Parameters of the RGBWW LEDs and light environment. (**a**) The normalized SPDs of red, green, blue, warm white, and cool white LEDs. (**b**) Illuminance and CCT, (**c**) CAF and CRI of the light environment throughout the day. (**d**) Time-dependent duty cycles of five channels throughout the day. (The figure was generated by Origin 2018, URL: https://www.originlab.com/).

The light sources were selected based on the following factors. According to the Grassmann Color Law^55^, three monochromatic LEDs were selected, whose chromaticity coordinates were located on the vertex of CIE 1931 color space. The peak wavelengths of three monochromatic LEDs were 454, 513, and 632 nm, respectively. Two phosphor-coated white light LEDs at different CCTs could increase the continuity of spectrum, color fidelity, and efficiency of mixed white light^29^. The CCTs of white LEDs were 2937 and 6733 K. Their spectral power distributions (SPDs) were shown in Fig. 6a.

The spectrum and some parameters of the natural daylight were measured from sunrise to sunset on May 31st, 2018 in Beijing^35^. We obtained time-dependent visual and non-visual parameters of the natural daylight, including illuminance, CCT, and CAF. The CCT of natural daylight ranged from 3508 to 5744 K and the CAF ranged from 0.51 to 0.86. The tunability of CCT was 1.64 times and it was 1.69 times for CAF. The CCT and CAF were associated with non-visual effects on humans, such as rhythm, emotion, alertness, and cognitive performance^56^, so we considered the periodic CCT and CAF as the zeitgebers to entrain the human biological rhythm. To enhance the circadian effects, the light sources in the experiment were designed to provide properly higher circadian tunability^34^ because the higher CCT and CAF corresponded to blue-enriched light, which entrained the circadian rhythm and promoted work performances during the day^5,13^, while the lower CCT and CAF at night meant less melatonin suppression^4,17^. Therefore, during the day (5:30 to 19:40), the variation ranges of CCT and CAF were 2680 to 7314 K and 0.32 to 0.96, respectively. The tunability of CCT and CAF increased to 2.80 and 3.00 times, respectively. At night, the CCT and CAF kept constants at approximately 2700 K and 0.32. Figure 6b and 6c show the time-dependent CCT and CAF throughout the day. The illuminance was designed to range from approximately 200 to 700 lx on the working table^56^, as shown in Fig. 6b. The color rendering index (CRI) was associated with visual comfort and non-visual performance of mood and rhythm^29^. The high CRI target was set to be above 90.0 during the day and above 95.0 at night. The experimental data of CRI are shown in Fig. 6c.

Based on the simulation targets and the SPDs of the RGBWW LEDs, an algorithm was applied to calculate the time-dependent cycle duties of five channels^35^. The chromaticity coordinates of the mixed white light were designed to fix exactly on the Planckian locus, to provide better visual comfort^57,58^. The time-dependent cycle duties of five channels are shown in Fig. 6d. The dark area corresponds to the nighttime when the duty cycles of five channels kept constants. The white area corresponds to the daytime (5:30 to 19:40) when the duty cycles of five LEDs changed to emit dynamic daylight-like light. The variation of cycle duties was less than 2% in one minute, indicating the slight change rate of light environment was not aware by humans.

### Biological effects measurement

**Figure 7:**
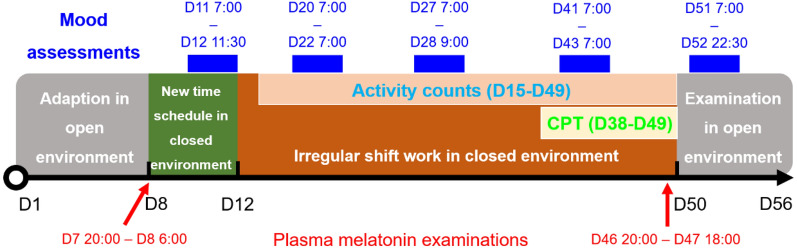
The experimental procedure and times of biological measurements from D1 to D56 (The figure was generated by PowerPoint 2019, URL: https://www.microsoftstore.com.cn/software/office).

Several physiological and behavioral measurements were performed during the experiment, including plasma melatonin, activity counts, CPT, and VAS on mood status, as shown in Fig. 7. Plasma melatonin was measured two times to assess the subjects’ physiological rhythm before and after the 38-day shift work, including baseline and terminal examinations. The baseline examination was from 20:00 on D7 to 6:00 on D8, which was at the beginning of living in the closed environment. The terminal examination was from 20:00 on D46 to 18:00 on D47, which was at the end of shift work. An indwelling intravenous catheter was placed in the forearm veins of subjects before 20:00 when subjects were awake. The blood samples were collected via the indwelling intravenous catheter at 20:00, 22:00, 0:00, 2:00, 4:00, and 6:00 in the baseline examination. In the terminal examination, the blood sample collections were extended to 8:00, 10:00, 12:00, 14:00, 16:00, and 18:00 of next day. The times of collecting blood samples were based on the time zone in the closed environment. In the baseline examination when subjects did not perform shift work, subjects were asleep when collecting blood samples at night, while in the terminal examination, two of them were asleep and one was working at night. At night, we collected the blood samples under the dim red light, to avoid waking up the subjects and disturbing their rhythms. The plasma melatonin concentration was determined by liquid chromatography-mass spectrum (LC–MS)^59^.

The activity counts were recorded by the wrist-activity monitors (Actiwatch, Philips Respironics) every minute. The monitors had an internal accelerometer to detect movement. The activity counts reflected the activity level and behavioral rhythm. The complete data was recorded from D15 to D49 because of devices trial and training for subjects.

CPT was used to measure the subjects’ sustained attention and cognitive performance^5,6^. There were 150 visual stimuli with 20% target stimuli in each test. The visual stimuli were the random numbers and two certain numbers were set as target stimuli. The position, size, and color of the number were constant. Subjects seated and performed the CPTs on the working table. They were asked to press the space bar as soon as the target stimuli appeared on the pad screen. Each visual stimulus presented 150 ms with an interval of 500 ms between two stimuli. The duration of each test was less than 2 min. During the daytime, the pad screen was in the normal mode and it changed into night mode during the nighttime. CPTs were performed in the first 10 min and the last 10 min of working segments. Subjects woke up 15 to 30 min before the working segments. Therefore, the CPTs were usually conducted 20 to 40 min after waking up to decrease the impact of sleep inertia^48^. We analyzed the data of CPTs on the last stage of the experiment (D38-D49) when subjects had engaged in shift work for 27 days.

Mood status was evaluated by a 100 mm bipolar VAS, including calmness/excitement, sleepiness/alertness, and happiness/sadness^46^. Subjects were required to assess their mood on the working table in work area. The VAS on mood assessments were performed in five sessions during the experiment. The first session was before shift work (D11 7:00–D12 11:30). The last session was after shift work (D51 7:00–D52 22:30). Another three sessions were during consecutive shift work in the closed environment (D20 7:00–D22 7:00, D27 7:00–D28 9:00, D41 7:00–D43 7:00). The interval between the assessments in each session was usually two hours, and the longest interval was four hours. For the three subjects, we performed more than 37 mood assessments in each session and the total number of mood assessments was 255 in five sessions.

### Statistical analysis

The interval of melatonin measurements was 2 h, so we obtained discrete melatonin concentrations in each measurement. We used cosine functions to fit these discrete data points and figured out some significant characteristics of plasma melatonin. The peak melatonin times and peak melatonin concentrations in the two examinations reflected the phase and strength of physiological rhythm, respectively^41^. We used paired-sample two-tailed t-test to evaluate whether the difference was significant between the two examinations. Also, 10 pg/ml was regarded as a threshold and the time when plasma melatonin exceeded 10 pg/ml was the melatonin onset time^41^. The SD of the three subjects’ onset times could reflect the time difference of melatonin secretion.

As for activity counts and behavioral rhythm, we calculated three nonparametric variables to analyze actigraphy data during the experiment, including interdaily stability (IS), intradaily variability (IV), and relative amplitude (RA)^45^. The IS describes the invariability of activity counts between the days, which represents the strength of coupling of the rhythm to the stable environmental zeitgebers. The IV describes the fragmentation of the rhythm, which reflects the frequency and extent of transitions between rest and activity. The RA is calculated from the most active 10 h and the least active 5 h in one day, which indicates the relative strength of the variation of activity counts^45^. People are supposed to be active during the daytime (most active 10 h) and sleep soundly at night (least active 5 h), so RA is assigned a favorable parameter^60^. To quantitatively describe the behavioral rhythm during the experiment, we compared the IS, IV, and RA of the first three days (D15–D17) with the last three days (D47–D49), and also compared these parameters of the first 6 days (D15–D20) with the last 6 days (D44–D49). The Wilcoxon signed-rank test was adopted to examine the significance of difference.

The number of correct responses and response time in the CPTs are the significant parameters to reflect the cognitive performance^5,6^. The responses to the target stimuli in 550 ms are the correct responses. The response time is the average response time in the correct responses in each test. Working between 8 am and 6 pm is regarded as day work, while working before 8 am or after 6 pm is regarded as night work^8^. We compared the number of correct responses and response time of the tests before and after the working segment. We also compared these two parameters at day work and night work. The significance of difference between before work and after work was examined by paired-sample two-tailed t-test, while the difference between day work and night work was examined by two-tailed t-test.

We adopted rANOVA to examine the significance of difference between five sessions mood assessments of the three kinds of mood status. *p* < 0.05 was set as the significant difference. As for the descriptive statistics on three kinds of mood, the mean and SEM were calculated from all the data in each session for all subjects.

## Data Availability

The data used and/or analyzed during the current study are available from the corresponding authors on reasonable request at any time.
